# Pheochromocytoma presenting with chest pain, heart failure and elevated pancreatic enzymes

**DOI:** 10.1093/omcr/omae202

**Published:** 2025-03-20

**Authors:** Qinglang Li, Shiyao Cheng, Zhichong Chen, Xi Hong, Biyao Wang, Mao Ouyang

**Affiliations:** Department of Cardiology, The Sixth Affiliated Hospital, Sun Yat-sen University, 26 Yuancun Erheng Road, Guangzhou 510655, China; Biomedical Innovation Center, The Sixth Affiliated Hospital, Sun Yat-sen University, Changzhou, Huangpu, Guangzhou 510700, China; Department of Cardiology, The Sixth Affiliated Hospital, Sun Yat-sen University, 26 Yuancun Erheng Road, Guangzhou 510655, China; Biomedical Innovation Center, The Sixth Affiliated Hospital, Sun Yat-sen University, Changzhou, Huangpu, Guangzhou 510700, China; Department of Cardiology, The Sixth Affiliated Hospital, Sun Yat-sen University, 26 Yuancun Erheng Road, Guangzhou 510655, China; Biomedical Innovation Center, The Sixth Affiliated Hospital, Sun Yat-sen University, Changzhou, Huangpu, Guangzhou 510700, China; Department of Cardiology, The Sixth Affiliated Hospital, Sun Yat-sen University, 26 Yuancun Erheng Road, Guangzhou 510655, China; Biomedical Innovation Center, The Sixth Affiliated Hospital, Sun Yat-sen University, Changzhou, Huangpu, Guangzhou 510700, China; Department of Cardiology, The Sixth Affiliated Hospital, Sun Yat-sen University, 26 Yuancun Erheng Road, Guangzhou 510655, China; Biomedical Innovation Center, The Sixth Affiliated Hospital, Sun Yat-sen University, Changzhou, Huangpu, Guangzhou 510700, China; Department of Cardiology, The Sixth Affiliated Hospital, Sun Yat-sen University, 26 Yuancun Erheng Road, Guangzhou 510655, China; Biomedical Innovation Center, The Sixth Affiliated Hospital, Sun Yat-sen University, Changzhou, Huangpu, Guangzhou 510700, China

**Keywords:** pheochromocytoma, chest pain, heart failure, hyperamylasemia, hyperlipasemia

## Abstract

Pheochromocytoma can present with various clinical manifestations and potentially mislead physicians by mimicking other conditions. Here we report a patient presenting with acute chest pain, de Winter electrocardiogram (ECG) changes, and elevated troponin I; the patient was initially diagnosed with acute myocardial infarction, but coronary angiography revealed no significant stenosis. He experienced heart failure symptoms with elevated serum amylase, lipase, and catecholamines. Further examination confirmed the diagnosis of pheochromocytoma, the patient later underwent tumor resection and recovered well. This case highlights the importance of rapid recognition and management of pheochromocytoma.

## Introduction

Pheochromocytoma is relatively rare neuroendocrine tumor originating from chromaffin cells in the adrenal medulla [1]. The age-standardized incidence rate of pheochromocytoma was 0.46 per 100 000 person-years [2], and it mainly occurs between ages 30–50 [3]. Pheochromocytoma can be asymptomatic until they secrete catecholamines like dopamine, epinephrine, and norepinephrine. The de Winter electrocardiogram (ECG) pattern is usually observed in severe coronary atherosclerotic disease [4], while hyperamylasemia and hyperlipasemia are often associated with pancreatitis. Pheochromocytoma presenting with de Winter ECG pattern and pancreatitis-independent hyperamylasemia and hyperlipasemia is rare.

## Case report

**Figure 1:**
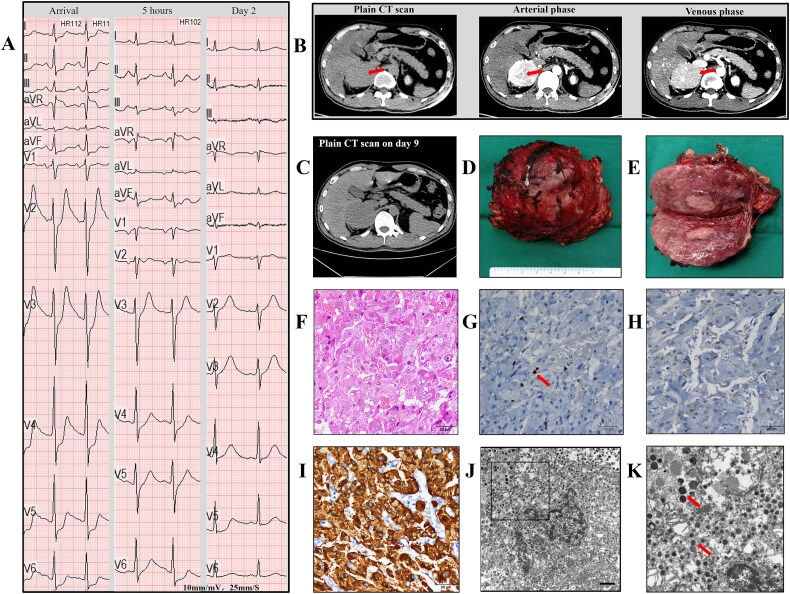
Electrocardiogram (ECG), computed tomography (CT) images and pathologic characteristics of pheochromocytoma. (A) 12-lead ECG on arrival, 5 hours later and day 2. (B) CT scan revealing a normal pancreas and a right adrenal mass (arrow) during the plain scan, arterial phase and venous phase. (C) Plain CT scan on day 9. (D) Gross appearance of the resected adrenal tumor, approximately 7 cm in length. (E) Cut surface revealing a fleshy tumor with areas of hemorrhage. (F) Haematoxylin-eosin (H&E)-stained section showing disordered and pleomorphic tumor cells. (G) Immunohistochemical staining for the Ki-67 proliferation marker was positive in 1% of the cells (arrow). (H) Immunohistochemical staining for S-100 protein was negative. (I) Immunohistochemistry for synaptophysin (Syn) was diffusely positive. (J) an electron microscopic image of the adrenal tumor. (K) Higher magnification of the area denoted by the black box in J, with arrows indicating secretory granules. All scale bars: 20 μm.

A 55-year-old man presented to the emergency department with a 3-h history of crushing chest pain. He had no history of hypertension, diabetes, or alcohol abuse. His vital signs were: blood pressure 184/110 mmHg, heart rate 124 beats/min, respiratory rate 25 breaths/min, oxygen saturation 98% on room air, and a body temperature 36.3°C. Electrocardiogram (ECG) showed de Winter pattern (Fig. 1A), with rapid troponin I at 0.44 ng/mL (normal range 0–0.30). The patient was diagnosed with acute myocardial infarction and received 300 mg aspirin, 300 mg clopidogrel. Additionally, glyceryl trinitrate was administered intravenously to control blood pressure and promote coronary vasodilation. Subsequently, chest pain improved and blood pressure decreased to normal. However, cardiac catheterization showed no coronary artery stenosis. Meanwhile, laboratory tests revealed markedly elevated serum amylase (7021.27 IU/L; normal range 15.00–135.00) and lipase (175.01 IU/L; normal range 0–66.00). A contrast-enhanced aortic computed tomography (CT) angiography scan was then performed to assess for potential aortic dissection, but no sign of aortic dissection or pancreatitis was found. However, a 74 × 64 × 78-mm tumor was found in the right adrenal area with well-demarcated margins and arterial phase hyperenhancement (Fig. 1B) without signs of metastasis. The patient later developed a worsening heart failure presentation, including diffuse lung crackling and oxygen saturation of 79% on room air. NT-proBNP was significantly elevated at 3949.80 pg/mL (normal range 0–300.00). Arterial blood gas analysis revealed a low oxygen index of 198.01 mmHg (normal range 400.00–500.00). Echocardiography revealed interventricular septal and posterior wall motion abnormalities, with an ejection fraction of 48%. Morphine, furosemide, deslanoside, and high-flow nasal cannula oxygen therapy were administered, resulting in resolution of heart failure symptoms. NT-proBNP and oxygen index normalized after treatment (Supplemental Table 1), the ejection fraction was 68.4% on day 9.

Considering the paroxysms of hypertension, tachycardia, and the adrenal tumor, pheochromocytoma was suspected. Catecholamines and their metabolites were measured and all were elevated (Supplemental Table 1). The patient underwent tumor resection, with histopathology confirming pheochromocytoma (Fig. 1D–K). Post-surgery, the patient recovered well and was discharged, the serum amylase and lipase level decreased to normal gradually. During a one-year follow-up period, the patient reported no chest pain, abdominal pain, palpitations or hypertension.

## Discussion

The typical presentations of pheochromocytoma include paroxysmal hypertension and adrenergic symptoms like palpitations, sweating, and headache, however, the excess catecholamines secretion might induce other atypical presentations. The patient initially presented with chest pain, elevated troponin I and de Winter ECG change which strongly suggested acute myocardial infarction, but coronary angiography revealed no significant stenosis in this case, it’s believed that excessive catecholamine release caused coronary artery vasospasm, leading to chest pain and subsequent heart failure, similar with previous reports [5, 6], Notably, this course was usually reversible. In this case, the patient's ECG showed attenuated ST-segment depression 5 h later and no ischemic changes on day 2 (Fig. 1A). Additionally, there were no further episodes of worsening heart failure after treatment.

While pancreatitis is a common cause of elevated amylase and lipase, the patient did not report abdominal pain, and CT scans showed a normal pancreas on arrival and day 9 (Fig. 1B–C); therefore, pancreatitis was excluded. The liver plays an important role in amylase and lipase elimination, so liver function impairment may induce amylase and lipase elevation [7, 8]; however, liver function was normal in this case (Supplemental Table 1). Amylase and lipase are not only secreted by the pancreas, but also by other tissues, conditions like hypoxia, ischemia and trauma can trigger amylase and lipase release from non-pancreatic tissues, such as the lung, and intestinal tract [8].

Moreover, a proportion of amylase is eliminated through the kidneys [9]; thus, any situations that hamper this process may induce elevated pancreatic enzymes. The patient’s renal function was initially impaired with serum creatinine of 236.4 μmol/L (normal range 44.0–133.0) on arrival. The impaired renal function might have been related to vasoconstriction and hypoperfusion in response to catecholamine release. His renal function improved, with serum creatinine decreasing to normal after treatment. The impaired renal function may therefore contribute to the elevated pancreatic enzymes.

Macroamylasemia is an unusual cause for hyperamylasemia, as amylase binds to immunoglobulins or hydroxyethyl starch to form macromolecular complexes, making it difficult to be eliminated by the kidneys [8]. However, the urine amylase was high (11862.20 IU/L, normal range 0–450.00) at arrival and the serum amylase level decreased to normal gradually. Besides, the amylase-to-creatinine clearance ratio (ACCR) was calculated. The calculation of ACCR requires normal renal function [10]. Therefore, we used laboratory results from day 6, when the serum creatinine had returned to the normal range. The ACCR was calculated as follows: (urine amylase of 1604.40 IU/L × serum creatinine of 106.43 μmol/L)/(serum amylase of 515.03 IU/L × urine creatinine of 7296.12 μmol/L) × 100. The ACCR is often < 1% in macroamylasemia cases [10], whereas it was 4.54% for this patient, making macroamylasemia an unlikely cause of the hyperamylasemia.

## Conclusion

The clinical presentations of pheochromocytoma are complex, it’s essential to consider pheochromocytoma in the differential diagnosis for patients experiencing paroxysmal hypertension and tachycardia, even when accompanied by atypical symptoms such as chest pain and elevated pancreatic enzymes.

## Supplementary Material

Supplemental_table_1_omae202
