# Supplementary material for: Pheochromocytoma presenting with chest pain, heart failure and elevated pancreatic enzymes
Source: Oxf Med Case Reports. 2025 Mar 20;2025(3):omae202. doi: 10.1093/omcr/omae202 (PMC11924377; doi:10.1093/omcr/omae202)
Supplement: Supplemental_table_1_omae202 [file supplemental_table_1_omae202.pdf]

Table 1. Laboratory test.

Time / Item	Arrival	4 hours	12 hours	Day 2	Day 3	Day 6	Day 12	Day 16	1 year	Reference
**Troponin I (ng/ml)**	**0.44**	**6.46**	—	**12.04**	**5.99**	**0.06**	—	—	—	**0.0-0.3**
**NT-proBNP (pg/ml)**	**3949.8**	**3251.3**	—	**3574.8**	**4044.7**	**4948.8**	—	**657.0**	—	**0-300**
**Amylase (IU/L)**	**7021.27**	**9624.03**	—	**2429.73**	**550.25**	**515.03**	**248.35**	—	**137.67**	**15.00-135.00**
**Lipase (IU/L)**	—	**175.01**	—	—	—	**936.26**	**168.49**	—	**12.54**	**0.00-66.00**
**Urine amylase (IU/L)**	—	**69.70**	—	**11862.20**	—	**1604.40**	—	—	—	**0.00-450.00**
**Creatinine (μmol/L)**	**236.4**	**261.65**	—	**146.95**	**119.52**	**106.43**	—	**94.17**	**91.64**	**44.00-133.00**
**ALT (IU/L)**	**57.07**	**50.48**	—	—	**34.17**	**32.75**	—	—	**26.31**	**0-40.00**
**AST (IU/L)**	**38.39**	**64.01**	—	—	**75.97**	**37.73**	—	—	**21.61**	**0-40.00**
**TBIL (μmol/L)**	**16.24**	**17.03**	—	—	—	**9.82**	—	—	**12.89**	**5.00-21.00**
**Urine creatinine (μmol/L)**	—	—	—	**19756.16**	—	**7296.12**	—	—	—	—
**Norepinephrine (pmol/L)**	—	—	**102756.0**	—	—	—	—	—	—	**414.0-4435.5**
**Epinephrine (pmol/L)**	—	—	**13499.0**	—	—	—	—	—	—	**≤ 605.4**
**Dopamine (pmol/L)**	—	—	**535.6**	—	—	—	—	—	—	**≤ 195.7**
**3-methoxytyramine (nmol/L)**	—	—	**0.65**	—	—	—	—	—	—	**<0.18**
**Metanephrine (nmol/L)**	—	—	**17.84**	—	—	—	—	—	—	**≤ 0.50**
**Normetanephrine (nmol/L)**	—	—	**>20.56**	—	—	—	—	—	—	**≤ 0.90**
**Urine VMA (mg/24h)**	—	—	—	**15.3**	—	—	—	—	—	**0.00-12.00**
**OI (mmHg)**	—	**198.1**	—	—	—	—	—	**424.4**	—	**400-500**

ALT, alanine aminotransferase; AST, aspartate aminotransferase; OI, oxygenation index; TBIL, total bilirubin; VMA, vanillylmandelic acid.
